# Grading and prognostic implications of tertiary lymphoid structures and CD155 expression in invasive breast carcinoma

**DOI:** 10.3389/fonc.2026.1895307

**Published:** 2026-10-30

**Authors:** Wenxin Yang, Yiming Li, Guiming Wang, Jinping Wang, Biling Zhong

**Affiliations:** Department of Pathology, Guangdong Second Provincial General Hospital, The Affiliated Guangdong Second Provincial General Hospital of Jinan University, Guangzhou, Guangdong, China

**Keywords:** breast invasive carcinoma, CD155, Ki-67, prognosis, recurrence, tertiary lymphoid structures (TLS), tumor immune microenvironment

## Abstract

**Objective:**

This study investigated the distribution and maturity of tertiary lymphoid structures (TLS) in invasive breast carcinoma tissue, as well as their prognostic value for postoperative recurrence.

**Methods:**

This study retrospectively collected tissue samples from 127 patients with invasive breast carcinoma at the Second People’s Hospital of Guangdong Province between January 2021 and December 2023. Immunohistochemistry was conducted to measure TLS and the expression of related proteins in invasive breast carcinoma and peritumoral tissues. Univariate and multivariate analyses were performed to determine the association between TLS and postoperative recurrence during the follow-up period. Receiver operating characteristic (ROC) curves were constructed to evaluate the discriminatory performance of TLS for postoperative recurrence.

**Results:**

The number and grade of TLS in invasive breast carcinoma tissue, as well as CD155 and Ki-67 expression, were markedly higher than those in peritumoral tissue. Compared with the non-recurrence group, patients in the recurrence group had a decreased number and grade of TLS in their tumor tissue and exhibited higher CD155 and Ki-67 expression intensity. Multivariate logistic regression analysis revealed that breast-conserving surgery, high CD155 expression, and high Ki-67 expression were independent positive predictors of postoperative recurrence, whereas a higher TLS grade was an independent negative predictor. TLS grade demonstrated moderate discriminatory performance, with an area under the ROC curve of 0.740, a sensitivity of 91.30%, and a specificity of 56.70%.

**Conclusion:**

TLS presence in invasive breast carcinoma tissue and its maturity level are negatively associated with recurrence risk, serving as a potential prognostic marker for recurrence in patients with invasive breast carcinoma.

## 1 Introduction

Breast cancer (BC) is one of the most prevalent malignant tumors among women, and its incidence and mortality rates have long ranked among the highest. According to the latest statistics from GLOBOCAN, the number of new BC cases continues to rise, making it a chief cause of cancer-related deaths among women worldwide. With the widespread adoption of early screening and advances in treatment modalities, the overall survival rate of BC patients has markedly improved. However, tumor recurrence and metastasis remain key factors affecting long-term survival. BC severely impairs patients’ quality of life and mental health (1). High medical costs place a heavy financial burden on patients’ families and the social healthcare system (2). Improving the prognosis for BC patients and providing more precise, personalized treatment have become top priorities in clinical practice and research. Tumor size, lymph node metastasis (LNM) status, histological grade, and the expression status of estrogen receptor (ER), progesterone receptor (PR), human epidermal growth factor receptor 2 (HER2), and Ki-67 are fundamental to BC classification and prognostic assessment (3). Among these, the mutation status of the tumor suppressor gene p53, as well as the expression levels of ER and PR, are associated with tumor proliferative activity, treatment sensitivity, and patient survival outcomes (3, 4). In recent years, tertiary lymphoid structures (TLS) in the tumor microenvironment (TME) have emerged as a promising prognostic predictor (5), and the immune components within the TME, particularly TLS, have garnered broad attention.

TLS refers to highly organized aggregates of lymphocytes that form ectopically in non-lymphoid tissues, such as tumors (5). TLS presence may indicate an antitumor immune response activated locally within the tumor. Detecting TLS via routine histopathological sections is cost-effective and convenient, making it feasible for clinical implementation and offering a new perspective for assessing the prognosis of BC. The prognostic value of TLS for BC patients remains controversial. Some studies believe that TLS acts as a protective factor, as the presence of mature TLS within BC tissue, along with higher levels of tumor-infiltrating lymphocytes, is associated with better response to neoadjuvant chemotherapy, lower recurrence risk, and longer overall survival (6, 7). Other studies, however, present a contrary view, suggesting that TLS may act as a risk factor. It has been hypothesized that immature or dysfunctional TLS may promote immunosuppression and tumor progression by recruiting regulatory T cells (Tregs) or generating a tumor-promoting inflammatory environment (8). Although TLS is generally associated with enhanced antitumor immune responses and favorable clinical outcomes in several malignancies, their prognostic significance may vary according to tumor type, spatial localization, cellular composition, and maturation status. In particular, mature TLS, containing organized B-cell follicles and germinal centers, may reflect a more coordinated local antitumor immune response, whereas the biological and prognostic significance of immature TLS remains unclear. Therefore, assessing TLS maturation, rather than TLS presence alone, may provide additional insights into the immune TME and clinical outcomes. These observations provide the rationale for evaluating both the presence and maturation status of TLS in invasive breast carcinoma. This study sought to systematically evaluate the presence and maturity of TLS in tumor and peritumoral tissues, as well as its correlation with conventional pathological indicators, through a retrospective analysis of clinical and pathological data and BC tissue samples from patients with invasive breast carcinoma, hoping to provide new theoretical support for prognostic assessment in BC.

## 2 Methods

### 2.1 Study population

We retrospectively gathered data on 127 patients with invasive breast carcinoma at the Second People’s Hospital of Guangdong Province between January 2021 and December 2023, including tumor and peritumoral tissue samples as well as clinical information. This study was approved by the Ethics Committee of the Second People’s Hospital of Guangdong Province (Ethics Approval No.: 2025-KY-KZ-224-01). Inclusion criteria covered: (1) Female patients with pathologically confirmed invasive breast carcinoma who underwent definitive breast surgery, including mastectomy or breast-conserving surgery; (2) Patients with clear tumor staging and complete clinical and pathological data; (3) No other malignant tumors; (4) Complete follow-up data. Exclusion criteria were as follows: (1) Missing follow-up data or clinical treatment records; (2) Other types of BC; (3) Unclear tumor staging; (4) Male patients. A total of 137 patients were initially screened. After exclusion of 10 patients because of incomplete clinical data (n = 3), incomplete follow-up data (n = 2), other malignancies (n = 3), or male sex (n = 2), 127 patients were finally included. The patient selection process is summarized in the flow diagram (**Supplementary Figure S1**).

Complete follow-up was defined as the availability of postoperative follow-up information regarding recurrence status from the date of surgery to the last documented follow-up. Patients without sufficient follow-up information to determine recurrence status were excluded from the analysis. No patients included in the final cohort were lost to follow-up. All histopathological and immunohistochemical assessments were performed on primary breast carcinoma specimens obtained during definitive surgery. Recurrent tumor specimens were not included in the pathological analysis.

### 2.2 Definition of follow-up and recurrence

Patients were followed from the date of definitive breast surgery to the date of the last documented follow-up. Postoperative recurrence was defined as the first documented occurrence of ipsilateral or contralateral breast recurrence, regional recurrence, or distant metastasis during the follow-up period. All-cause mortality was also recorded. However, no deaths occurred during the observed follow-up period. The median follow-up duration was 43 months.

### 2.3 Variables

Clinical data we collected covered age, clinical stage, tumor diameter, histological grade, LNM, distant metastasis, nerve or vascular invasion, histological type, degree of differentiation, treatment modality, and history of radiotherapy and chemotherapy. Additionally, the expression levels of Ki-67, CD4, CD8, CD20, ER, PR, CD155, p53, HER2, and EGFR were detected.

### 2.4 Immunohistochemical assay

Tumor tissue samples were fixed overnight in 10% formalin, followed by standard dehydration,clearing, and paraffin embedding. Slice thickness was set to 4 μm to ensure adequate antibodypenetration. The sections were dried in an oven at 60 °C for 1 hour, deparaffinized with xylene, and rehydrated using a series of alcohol solutions. Antigen retrieval was performed using an antigen retrieval solution, followed by microwave heating (95 °C, 20 minutes) and cooling to room temperature. Next, tissue sections were washed three times with phosphate-buffered saline (PBS) for 5 minutes each time to remove excess fixative and blocked with 3% H_2_O_2_ for 10 minutes. Then, drops of the appropriate primary antibody dilution were applied to the tissue sections overnight at 4 °C to ensure sufficient binding between the antibody and antigen. The primary antibodies used for immunohistochemistry, including their clones, manufacturers, catalogue numbers, and working dilutions, are summarized in **Supplementary Table S1**. The following day, the sections were washed three times with PBS for 5 minutes each time. Subsequently, an appropriate amount of horseradish peroxidase-labeled secondary antibody was added and incubated at room temperature for 30 minutes, followed by washing with PBS. The sections were treated with diaminobenzidine staining solution. Once the desired color intensity was achieved, the reaction was stopped with distilled water. After counterstaining with hematoxylin, the sections were dehydrated, cleared, and mounted, and protein expression was finally observed and recorded under a microscope. Throughout all procedural steps, a clean environment should be maintained to prevent potential contamination that could interfere with the results. The criteria for a positive result were as follows: the presence of distinct, uniformly distributed brown granular deposits in the cell nucleus or cytoplasm; or very prominent, dark-colored, and widely distributed granular deposits. The criteria for a negative result were as follows: the absence of brown granular deposits in the cell nucleus or cytoplasm; or the presence of faint, pale-colored, or unevenly distributed granular deposits.

### 2.5 Definition and grading of TLS

TLS was preliminarily identified using hematoxylin and eosin (H&E) staining, and TLS grading was conducted in conjunction with immunohistochemical markers. The diagnostic criteria for TLS involved clusters of lymphocytes in H&E sections and the presence of CD4-, CD8-, and CD20-positive T and B lymphocytes in immunohistochemical sections; and the presence of lymphocyte clusters (9).

As shown in **Figure 1**, TLS in invasive breast carcinoma was classified into four grades based on the spatial distribution of lymphocytes within the TLS. Grade 0: No TLS in the section. Grade 1: Early-stage TLS, manifesting solely as dense foci of lymphocyte clusters. Grade 2: Clusters of T-cell and B-cell areas characteristic of TLS, without germinal centers. Grade 3: In addition to the features of Grade 2, germinal centers are present (5). All H&E-stained slides were independently reviewed by two pathologists, who reassessed the presence and maturation grade of TLSs based on their morphological features. Particular attention was paid to the identification of germinal center formation. In cases where there were disagreements, a joint review was conducted to reach a consensus.

**Figure 1:**
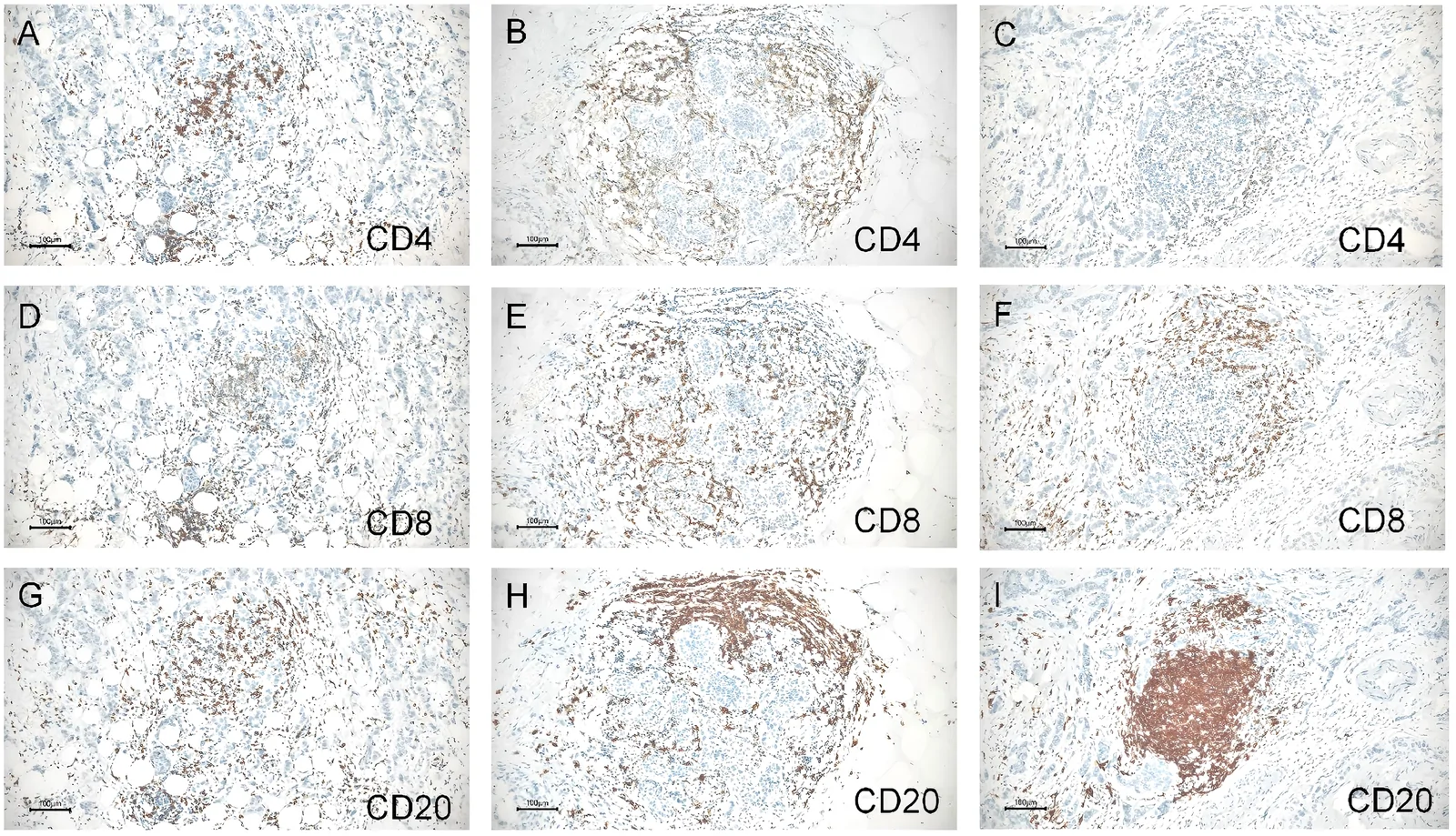
Morphological and immunophenotypic characteristics of tertiary lymphoid structures (TLS) of varying maturity grades in breast cancer tissue. TLS Grade 1 **(A, D, G)**, TLS Grade 2 **(B, E, H)**, TLS Grade 3 **(C, F, I)**; magnification ×200.

### 2.6 CD155 expression

CD155 expression in tumor cells was evaluated semi-quantitatively according to staining intensity: grade 0, no staining; grade 1, weak staining; grade 2, moderate staining; and grade 3, strong staining. Membranous and/or cytoplasmic staining of tumor cells was considered positive. Two pathologists independently evaluated the immunostained slides, and discrepancies were resolved by consensus.

### 2.7 Statistical methods

Statistical analyses were conducted in SPSS 26.0 and R 4.2.1 software. Quantitative data were examined using the Kolmogorov-Smirnov normality test; variables conforming to a normal distribution were reported as mean ± standard deviation, while those not conforming were reported as median (interquartile range). For data that were normally distributed and had homogeneous variances, the t-test was applied for comparisons; for data that did not meet these criteria, the Mann-Whitney U test (rank-sum test) was adopted for comparisons. Qualitative data were represented as case counts (percentages); comparisons were made using the χ² test or Fisher’s exact test. Spearman’s correlation coefficient was employed to analyze the correlations among variables. Multivariate logistic regression was leveraged to pinpoint independent risk factors for recurrence. Receiver operating characteristic (ROC) curves were utilized to evaluate the discriminatory performance of each indicator, and the area under the curve (AUC) was computed. P < 0.05 was considered statistically significant.

## 3 Results

### 3.1 Patient demographics

This study enrolled 127 patients with invasive breast carcinoma. Their demographic characteristics are outlined in **Table 1**. Their median age was 51 (45, 60) years; the majority had a history of childbirth (92.9%) and were premenopausal (n=97, 76.4%. The predominant pathological type was invasive carcinoma of no special type (NST) (94.5%), and the median tumor size was 2.50 cm. Regarding staging, stage T2 (56.7%) and lymph node-negative (N0, 46.5%) were the most common. The predominant differentiation grades were moderately differentiated (47.2%) and poorly differentiated (48.0%). The rates of axillary LNM and vascular invasion were 46.5% and 64.6%, respectively, but the rate of distant metastasis was low (2.4%). In addition, 81.1% of patients underwent radical surgery, 65.4% received postoperative chemotherapy, and only 24.4% underwent postoperative radiotherapy. The median CA15–3 level was 8.15, and the median total TLS count and grade were 3 and 1, respectively. During a median follow-up period of 43 months, the recurrence rate was 18.1% (23/127).

**Table 1:** Patient characteristics [M (P25, P75), (), n (%)].

Variables		Description
Age		51 (45, 60)
Childbirth	No	9 (7.10)
	Yes	118 (92.9)
Menopausal status		
	Premenopausal	97 (76.4)
	Postmenopausal	30 (23.6)
Tumor size		2.50 (1.80, 3.50)
Affected side	Left	61 (48.0)
	Right	66 (52.0)
Pathological type	Other special histological types	7 (5.5)
	invasive carcinoma of no special type (NST)	120 (94.5)
T	1	42 (33.1)
	2	72 (56.7)
	3	11 (8.70)
	4	2 (1.60)
N	0	59 (46.5)
	1	50 (39.4)
	2	11 (8.70)
	3	7 (5.50)
M	0	124 (97.6)
	1	3 (2.40)
Differentiation	Highly differentiated	6 (4.70)
	Moderately differentiated	60 (47.2)
	Poorly differentiated	61 (48.0)
Axillary lymph node metastasis	No	68 (53.5)
	Yes	59 (46.5)
Percentage of metastasis		15.4 (10.0, 37.5)
Perineural invasion	No	100 (78.7)
	Yes	27 (21.3)
Vascular invasion	No	45 (35.4)
	Yes	82 (64.6)
Distant metastasis	No	124 (97.6)
	Yes	3 (2.40)
Treatment methods	Curative	103 (81.1)
	Breast-conserving	24 (18.9)
Postoperative chemotherapy	No	44 (34.6)
	Yes	83 (65.4)
Postoperative radiotherapy	No	96 (75.6)
	Yes	31 (24.4)
Surgical modality	Mastectomy	103 (81.10)
	Breast-conserving surgery	24 (18.90)
CA153		8.15 (4.94, 12.56)
TLS total number		3 (1, 6)
TLS grade		1 (1, 3)
Postoperative recurrence during follow-up	No	104 (81.9)
	Yes	23 (18.1)
Time of recurrence		22.77 ± 17.09

### 3.2 Immunohistochemical markers and TLS presence in tumor and peritumoral tissue

A comparative analysis (**Table 2**) of the distribution of multiple immunohistochemical markers in tumor and peritumoral tissues revealed significantly higher TLS counts in tumor tissue (2 [0, 5]) than in peritumoral tissue (0 [0, 2]). The distribution of various lymphocyte subtypes (CD4, CD8, CD20) and the expression percentage of the proliferation marker KI67 were all markedly elevated in tumor tissue (all P < 0.001). The expression of hormone receptors ER and PR was also substantially higher in tumor tissue than in peritumoral tissue. Furthermore, the positive expression of CD155, P53 mutant, HER2 (1+ and 3+), and EGFR was more prevalent in tumor tissue (all P < 0.05).

**Table 2:** Distribution of tumor and peritumor parameters in patients [M (P25, P75), n (%)].

Parameters		Peritumor	Tumor	*Z/χ^2^*	*P value*
Number of cancerous and borderline TLS cases		0 (0, 2)	2 (0, 5)	-4.245	<0.001
Percentage of CD4-positive cells		5 (0, 10)	10 (1, 40)	-4.328	<0.001
Percentage of CD8-positive cells		5 (0, 10)	10 (5, 40)	-5.447	<0.001
Percentage of CD20-positive cells		5 (0, 10)	15 (5, 35)	-7.010	<0.001
Percentage of KI67-positive cells		2 (2, 5)	20 (15, 40)	-13.290	<0.001
Percentage of ER-positive cells		5 (5, 10)	80 (0, 90)	-6.682	<0.001
Percentage of PR-positive cells		5 (4, 8)	60 (0, 90)	-4.169	<0.001
CD155 expression	0	111 (87.4)	32 (25.2)	121.674	<0.001
	1	16 (12.6)	17 (13.4)		
	2	0 (0.0)	33 (26.0)		
	3	0 (0.0)	45 (35.4)		
P53 expression	Wild-type	126 (99.2)	71 (55.9)	68.426	<0.001
	Mutant	1 (0.8)	56 (44.1)		
HER2 expression	0	126 (99.2)	28 (22.0)	158.442	<0.001
	1	1 (0.8)	50 (39.4)		
	3	0 (0.0)	49 (38.6)		
EGFR expression	Negative	127 (100.0)	98 (77.2)	32.738	<0.001
	Positive	0 (0.0)	29 (22.8)		

### 3.3 Characteristics of patients with and without recurrence

**Table 3** presents the demographic and clinical characteristics of patients with invasive breast carcinoma who experienced postoperative recurrence versus those who did not experience recurrence during the follow-up period. Among multiple variables, vascular invasion, treatment modality, the total number of TLS, and TLS grade exhibited notable differences between the two groups (P < 0.05). Vascular invasion was more common in the recurrence group (82.0% vs. 60%), and a higher proportion of patients in this group underwent breast-conserving surgery (39.0% vs. 14.0%). The total number and grade of TLS were markedly higher in the non-recurrence group (P < 0.001). No notable differences were noted in other indicators, such as age, childbirth, menopause, tumor size, histological type, T/N staging, differentiation, axillary LNM, nerve invasion, or postoperative chemotherapy/radiotherapy. Among the nine patients who underwent breast-conserving surgery in the recurrence group, seven received postoperative radiotherapy, whereas two refused radiotherapy for family-related reasons.

**Table 3:** Comparison of demographic and clinical characteristics between patients with and without recurrence [M (P25, P75), n (%)].

Variables		Non-recurrence (n=104)	Recurrence (n=23)	*Z/χ^2^*	*P* value
Age		52 (45, 60)	48 (45, 60)	-1.046	0.295
Childbirth	No	7 (6.7)	2 (8.7)	0.000	1.000
	Yes	97 (93.3)	21 (91.3)		
Menopause	No	80 (76.9)	17 (73.9)	0.095	0.758
	Yes	24 (23.1)	6 (26.1)		
Tumor size		2.5 (1.8, 3.6)	2.5 (2, 3.5)	-0.712	0.476
Affected side	Left	49 (47.1)	12 (52.2)	0.193	0.660
	Right	55 (52.9)	11 (47.8)		
Histological type	Other special histological types	5 (4.8)	2 (8.7)	1.093	0.296
	Invasive carcinoma of no special type (NST)	99 (95.2)	21 (91.3)		
T	1	38 (36.0)	4 (17.0)	5.602	0.096
	2	55 (52.0)	17 (73.0)		
	3	10 (9.60)	1 (4.3)		
	4	1 (1.0)	1 (4.3)		
N	0	50 (48.0)	9 (39.)	1.955	0.582
	1	39 (37.0)	11 (47.)		
	2	10 (9.6)	1 (4.3)		
	3	5 (4.8)	2 (8.7)		
M	0	104 (100)	20 (87.0)	8.814	0.005
	1	0 (0.0)	3 (13.0)		
Differentiation	Highly differentiated	5 (4.8)	1 (4.3)	0.823	0.663
	Moderately differentiated	51 (49.0)	9 (39.0)		
	Poorly differentiated	48 (46.0)	13 (56.0)		
Axillary lymph node metastasis	No	58 (55.0)	10 (43.0)	1.144	0.285
	Yes	46 (44.0)	13 (56.0)		
Percentage of metastasis			6.67 (0, 40)	-1.527	0.127
Perineural invasion	No	83 (79.0)	17 (73.)	0.118	0.731
	Yes	21 (20.0)	6 (26.)		
Vascular invasion	No	41 (39.)	4 (17.0)	3.996	0.046
	Yes	63 (60.)	19 (82.0)		
Distant metastasis	No	104 (100)	20 (87.0)	8.814	0.005
	Yes	0 (0.0)	3 (13.0)		
Treatment methods	Curative	89 (85.0)	14 (60.0)	5.976	0.014
	Breast-conserving	15 (14.0)	9 (39.0)		
Postoperative chemotherapy	No	39 (37.0)	5 (21.0)	2.066	0.151
	Yes	65 (62.0)	18 (78.0)		
Postoperative radiotherapy	No	80 (76.0)	16 (69.0)	0.553	0.457
	Yes	24 (23.0)	7 (30.0)		
CA153		8.07 (4.71, 12.72)	8.56 (7.78, 12.56)	-0.660	0.509
TLS total number		4 (2, 6)	1 (0, 2)	-3.645	<0.001
TLS grade		2 (1, 3)	1 (0, 1)	-3.783	<0.001
Percentage of tumor cells		60 (50, 70)	60 (50, 60)	-0.737	0.461

The percentage of KI67-positive cells was markedly higher in the tumor tissue of patients who experienced recurrence (P = 0.025), and CD155 expression intensity was stronger in the recurrence group (91.3% were grade 3, P < 0.001). Additionally, the proportion of TLS in the tumor and borderline areas was greatly lower in the recurrence group than in the non-recurrence group (P < 0.001). In peritumoral tissue, the CD155 positivity rate was also markedly higher in the recurrence group than in the non-recurrence group (34.8% vs. 7.7%, P < 0.001). No notable differences were detected for most other immunohistochemical markers (PR, ER, P53, HER2, and EGFR) (**Table 4** and **5**).

**Table 4:** Comparison of marker expression in tumor tissues of patients with and without recurrence [M (P25, P75), n (%)].

Parameters		Non-recurrence	Recurrence	*Z/χ^2^*	*P* value
Number of cancerous and borderline TLS cases		3 (0, 6)	0 (0, 1)	-3.440	0.001
Percentage of CD4-positive cells		10 (1, 40)	10 (1, 40)	-0.464	0.643
Percentage of CD8-positive cells		10 (5, 40)	10 (2, 40)	-0.488	0.625
Percentage of CD20-positive cells		18 (5, 38)	10 (5, 20)	-0.779	0.436
Percentage of KI67-positive cells		20 (10, 40)	30 (20, 50)	-2.249	0.025
Percentage of ER-positive cells		80 (2, 90)	50 (0, 90)	-1.509	0.131
Percentage of PR-positive cells		75 (0, 90)	0 (0, 85)	-1.512	0.130
CD155 expression	0	32 (30.8)	0 (0.00)	36.374	<0.001
	1	17 (16.3)	0 (0.00)		
	2	31 (29.8)	2 (8.70)		
	3	24 (23.1)	21 (91.3)		
P53 expression	Wild-type	62 (59.6)	9 (39.1)	3.206	0.073
	Mutant	42 (40.4)	14 (60.9)		
HER2 expression	0	20 (19.2)	8 (34.8)	2.7	0.259
	1	42 (40.4)	8 (34.8)		
	3	42 (40.4)	7 (30.4)		
EGFR expression	Negative	84 (80.8)	14 (60.9)	4.233	0.054
	Positive	20 (19.2)	9 (39.1)		

**Table 5:** Comparison of marker expression in peritumoral tissue between patients with and without recurrence [M (P25, P75), n (%)].

Parameters		Non-recurrence	Recurrence	*Z/χ^2^*	*P value*
Number of cancerous and borderline TLS cases		0 (0, 2)	1 (0, 2)	-0.884	0.377
Percentage of CD4-positive cells		5 (0, 10)	5 (0, 10)	-0.356	0.722
Percentage of CD8-positive cells		5 (0, 10)	5 (0, 10)	-0.411	0.681
Percentage of CD20-positive cells		5 (1, 10)	5 (0, 5)	-0.698	0.485
Percentage of KI67-positive cells		2 (2, 5)	2 (2, 5)	-0.020	0.984
Percentage of ER-positive cells		6 (5, 10)	5 (0, 8)	-1.735	0.083
Percentage of PR-positive cells		5 (5, 8)	5 (0, 5)	-2.192	0.028
CD155 expression	0	96 (92.3)	15 (65.2)	10.213	0.001
	1	8 (7.70)	8 (34.8)		
P53 expression	Wild-type	103 (99.0)	23 (100.0)	0.000	1.000
	Mutant	1 (1.00)	0 (0.00)		
HER2 expression	0	103 (99.0)	23 (100.0)	0.000	1.00
	1	1 (1.00)	0 (0.00)		
EGFR expression	Negative	104 (100.0)	23 (100.0)	0.000	1.000
	Positive	0 (0.00)	0 (0.00)		

### 3.4 Analysis of recurrence-related factors

Significant associations were found between several factors and postoperative recurrence during the follow-up period (**Table 6**). Among these, CD155 expression showed a moderate positive association with recurrence (correlation coefficient = 0.503, P < 0.001). Most other indicators showed weak associations, including M staging, distant metastasis, treatment modality, total number of TLS, TLS grade, number of cancerous and borderline TLS, percentage of KI67 expression, EGFR expression, peritumoral CD155 expression, and percentage of peritumoral PR expression (all P < 0.05). Notably, all TLS-related indicators (total number of TLS, TLS grade, and cancerous and borderline TLSs) were negatively associated with recurrence.

**Table 6:** Analysis of factors associated with postoperative recurrence during follow-up.

Factors	Correlation coefficient	P value
M	0.331	<0.001
Vascular invasion	0.177	0.046
Distant metastasis	0.331	<0.001
Treatment modality	0.243	0.006
Total number of TLS	-0.325	<0.001
TLS grade	-0.337	<0.001
Number of cancerous and borderline TLS	-0.306	<0.001
CD155 expression	0.503	<0.001
Percentage of KI67 expression	0.200	0.024
EGFR expression	0.183	0.040
CD155 expression (peritumoral)	0.314	<0.001
Percentage of PR expression (peritumoral)	-0.195	0.028

**Table 7** presents a multivariate logistic regression analysis, with treatment modality, vascular invasion, total number of TLS in tumor tissue, TLS grade, CD155 expression, and KI67 expression percentage as independent variables, and recurrence status as the dependent variable. It revealed that treatment modality (breast-conserving surgery), CD155 expression, and KI67 expression percentage were independent positive predictors of recurrence (OR > 1). Breast-conserving surgery was associated with higher odds of postoperative recurrence in this cohort (OR = 10.069). In contrast, TLS grade was a negative predictor (OR = 0.352), indicating that a higher grade (more mature) of TLS was associated with a lower recurrence risk.

**Table 7:** Multivariate logistic regression analysis of factors associated with patient recurrence.

Variables	*B*	*SD*	*Wald*	*P value*	*OR(95%CI)*
Treatment modality (breast-conserving)	2.309	0.688	11.268	0.001	10.069 (2.614-38.779)
TLS grade	-1.045	0.305	11.761	0.001	0.352 (0.194-0.639)
CD155 expression (Grade 1)	1.995	0.692	8.319	0.004	7.350 (1.895-28.508)
Percentage of KI67 expression	0.028	0.013	4.298	0.038	1.028 (1.002-1.056)

### 3.5 Clinical value of TLS in the recurrence of invasive breast carcinoma

**Figure 2** and **Table 8** present the ROC curve evaluation results for each associated indicator. Among individual indicators, TLS grade demonstrated moderate discriminatory ability for postoperative recurrence, with an AUC of 0.740. The optimal cut-off value of TLS grade was 1.5, as determined by the maximum Youden index (0.480), with a sensitivity of 91.30% and a specificity of 56.70%. This cut-off value separated lower TLS maturation grades (grades 0–1) from higher TLS maturation grades (grades 2–3).

**Figure 2:**
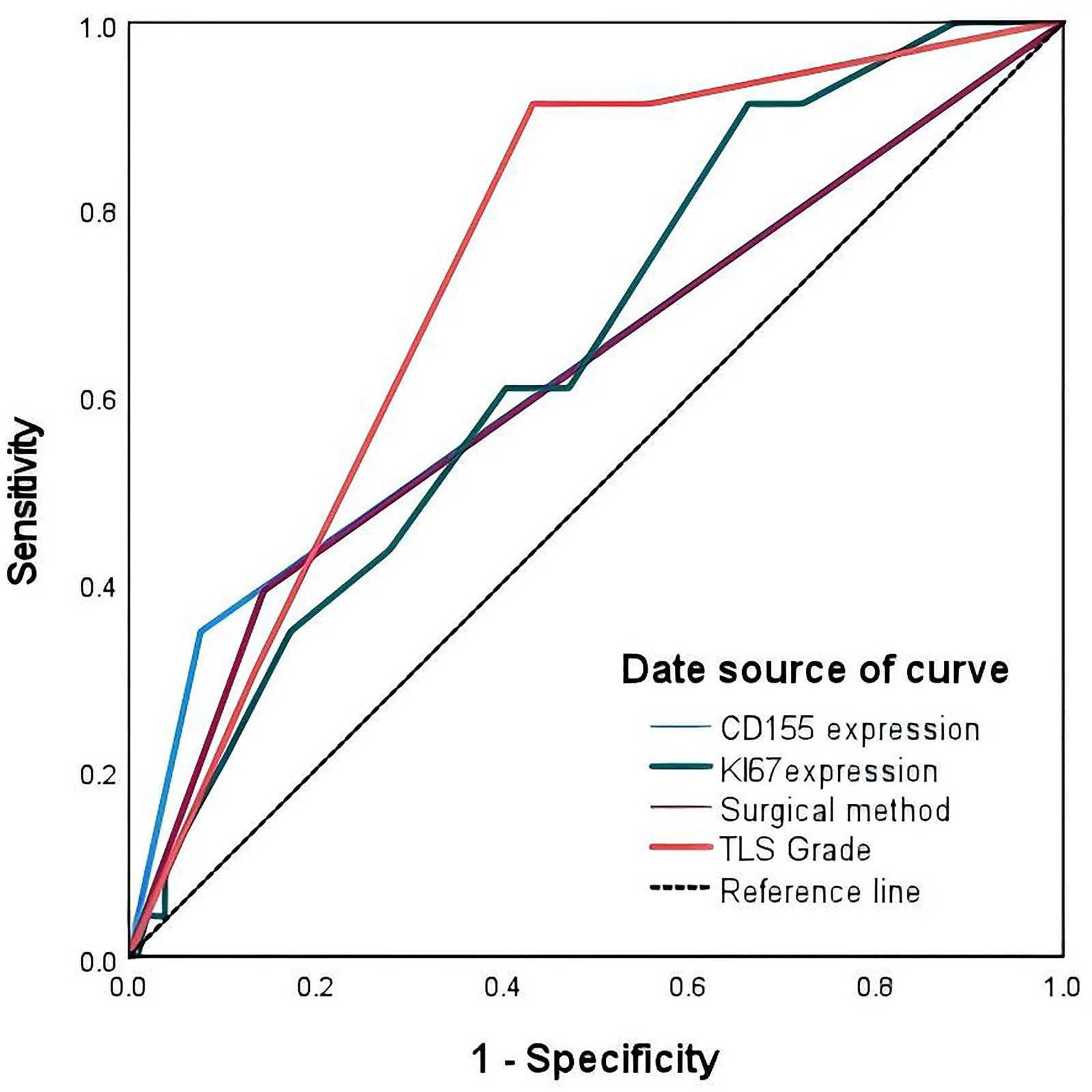
ROC curves for individual predictors and a combined model predicting postoperative recurrence of breast cancer.

**Table 8:** Evaluation of ROC curves.

Variables	AUC	Sensitivity	Specificity	Youden’s index	Cut-off value
Treatment modality	0.624 (0.487-0.760)	39.10	85.60	0.247	0.5
CD155 expression	0.635 (0.497-0.774)	34.80	92.30	0.271	0.5
Percentage of KI67 expression	0.649 (0.533-0.765)	91.30	33.70	0.250	17.5
TLS grade	0.740 (0.639-0.840)	91.30	56.70	0.480	1.5

## 4 Discussion

This study systematically evaluated the presence and maturity of TLS, as well as its associations with clinical and pathological indicators and prognosis, through a retrospective analysis of clinical and pathological data of 127 patients with invasive breast carcinoma. The results indicated that the quantity and grade of TLS in tumor tissue were considerably higher than in peritumoral tissue and were associated with infiltration of various immune cells and elevated expression of proliferation markers. Multivariate analysis indicated that breast-conserving surgery, high CD155 expression, and high KI67 expression were independent positive predictors of recurrence, whereas a higher TLS grade was a negative predictor, suggesting that TLS grading holds potential clinical utility in risk assessment. Notably, breast-conserving surgery was associated with higher odds of postoperative recurrence in our cohort (OR = 10.069). However, this finding should be interpreted with caution. The relatively small sample size and uneven distribution of patients between surgical groups may have resulted in an unstable effect estimate. In addition, the single-center retrospective design may have introduced selection bias and residual confounding. Therefore, this association should not be interpreted as evidence that breast-conserving surgery itself increases the risk of postoperative recurrence. Further larger, multicenter cohorts are warranted for validation.

In the recurrence group, the incidence of vascular invasion and the proportion of breast-conserving surgery were higher, whereas the total number and grade of TLS were markedly lower. TLS was negatively associated with recurrence. Regarding the role of TLS, our findings demonstrated an association between higher TLS maturation grades and a lower risk of postoperative recurrence. The ROC-derived cut-off of 1.5 suggests that TLS grades 0–1 and grades 2–3 may represent lower- and higher-maturation groups, respectively, with potential value for postoperative recurrence-risk stratification. However, given the moderate discriminatory performance (AUC = 0.740) and limited specificity (56.70%), this cut-off value should be considered exploratory and requires validation in independent cohorts before clinical application. One possible explanation is that mature TLS may contribute to a coordinated local antitumor immune response through the recruitment and activation of T and B lymphocytes. However, this potential mechanism remains hypothetical, as the present study provides correlative clinicopathological evidence rather than direct functional or spatial-phenotypic evidence. Higher TLS maturation grades were associated with a lower risk of postoperative recurrence in our cohort. One possible explanation is that more highly organized TLSs may be associated with a more coordinated local antitumor immune response involving T- and B-cell compartments. However, morphological maturation of TLSs does not necessarily indicate their functional immune status. Therefore, the biological mechanisms underlying the association between TLS maturation and recurrence observed in this study need further investigation. These findings are consistent with those reported by Cabrita et al. (6) and Helmink et al. (7) in melanoma, as well as the conclusions observed by Martinet et al. (8) in BC, which demonstrated a link between TLS and a high proportion of endothelial microvessels as well as a favorable prognosis. Figenschau et al. (9) similarly reported an association of TLS with higher tumor grade and better prognosis in primary BC, further supporting our findings. However, in contrast to the conclusion proposed by Posch et al. (10) that immature TLS may promote recurrence in early-stage non-small cell lung cancer, the TLS scoring system proposed by Rakaee et al. (11) in non-small cell lung cancer confirmed that mature TLS was closely associated with longer recurrence-free survival, emphasizing the clinical significance of assessing TLS maturity. Overall, TLS function may be dependent on tumor type and maturity. This study did not find a significant link between TLS and time to recurrence, which may be attributed to the heterogeneity of TLS functional states or the limited sample size. By quantifying TLS maturity through a grading system, this study provides structural-level evidence for its protective role (12, 13).

Furthermore, high CD155 expression in both tumor and peritumoral tissues was markedly associated with recurrence. As an immunoregulatory molecule, CD155 was identified in this study as a strong predictor of recurrence, with significantly elevated expression levels observed particularly in the recurrence group. This finding aligns with the mechanism by which CD155 impairs T-cell function by binding to inhibitory receptors, such as TIGIT (14). Furthermore, tumor cells can facilitate immunosuppression by altering microenvironmental signaling (15, 16), and CD155 may be one of the key molecules involved in such signaling. The innovation of this study lies in the simultaneous analysis of CD155 expression in tumor and peritumoral tissues, revealing that it is associated with recurrence in both regions. This suggests that CD155 may act as a cross-regional immunosuppressive signal, providing a new perspective for understanding its mechanism of promoting recurrence.

This study revealed a potential association of ER and PR expression with TLS. ER and PR expression levels were considerably higher in tumor tissue than in peritumoral tissue, and the number of TLS was also markedly augmented in tumor tissue, suggesting that TLS may be more active in the hormone receptor-positive TME. Higher levels of ER and PR were detected in the non-recurrence group, along with higher TLS grades. This suggests that ER- and PR-positive BC may provide a TME more conducive to TLS maturation and function. This study did not identify a clear link between TLS and HER2 status or P53 mutations in the available data. This may imply that TLS generation and function are independent of some classic oncogenic pathways and rely more on the local immune microenvironment. In this study, histological type was not significantly associated with postoperative recurrence. However, invasive carcinoma of NST accounted for most cases in our cohort, while other histological subtypes were relatively uncommon. Therefore, the limited number of non-NST cases restricts further subgroup analysis, and the potential prognostic impact of specific histological subtypes requires validation in larger cohorts. Postoperative chemotherapy and radiotherapy did not differ significantly between the recurrence and non-recurrence groups. However, among the nine patients who underwent breast-conserving surgery in the recurrence group, two refused postoperative radiotherapy for family-related reasons. The absence of radiotherapy in these patients may have contributed to the observed association between breast-conserving surgery and postoperative recurrence. Histological type and postoperative chemotherapy/radiotherapy were not included in the multivariate model. Nevertheless, potential residual confounding related to postoperative treatment cannot be excluded, and this association should be interpreted cautiously.

This study has certain limitations. First, the retrospective, single-center design and exclusion of patients with incomplete follow-up may have introduced selection bias. Although TLS maturation was independently reassessed by two pathologists based on histomorphological features, morphological maturation may not fully reflect the functional immune status of TLSs. Second, the relatively small sample size and the single-center retrospective study may introduce selection bias and recall bias. Furthermore, although TLS grading was associated with recurrence, the AUC was not particularly high. This may be due to the small sample size or the limited prognostic ability of TLS for recurrence. There is currently no unified international consensus standard for TLS assessment. In addition, the relatively small number of patients undergoing breast-conserving surgery and the uneven distribution of surgical modalities may have affected the stability of the estimated association between surgical modality and postoperative recurrence. Further validation in larger, multicenter cohorts with more balanced surgical groups is warranted. Although this study adopted a widely recognized grading method (5), caution is warranted regarding potential discrepancies in interpretation among different research centers (17). To address these limitations, future multicenter, large-scale prospective trials are required to further substantiate the generalizability of this model, combined with more in-depth investigations into molecular mechanisms, such as utilizing multiplex fluorescence staining to elucidate the cellular composition and spatial distribution within the TLS (18, 19). Of note, the TLS has recently been validated as a prognostic biomarker for the efficacy of immunotherapy in various solid tumors (17, 20). Future studies could further examine whether TLS grading similarly influences BC patients’ response to immune checkpoint inhibitors, thereby expanding its clinical utility. In summary, this study confirms the potential protective role of TLS in invasive breast carcinoma. It may serve as a new indicator for prognostic assessment and lays the foundation for further exploration of the interaction mechanisms between TLS and CD155 in the immune TME.

## Data Availability

The raw data supporting the conclusions of this article will be made available by the authors, without undue reservation.

## Supplementary material

Supplementary Table 1 Primary antibodies used for immunohistochemistry.

Supplementary Figure 1 Flow diagram of patient selection. A total of 137 patients were initially screened. Ten patients were excluded because of incomplete clinical data (n = 3), incomplete follow-up data (n = 2), other malignancies (n = 3), or male sex (n = 2), resulting in a final study cohort of 127 patients.
