# Supplementary material for: Grading and prognostic implications of tertiary lymphoid structures and CD155 expression in invasive breast carcinoma
Source: Front Oncol. 2026 Oct 30;16:1895307. doi: 10.3389/fonc.2026.1895307 (PMC13759285; doi:10.3389/fonc.2026.1895307)
Supplement: Supplementary file 1 [file Table1.docx]

**Supplementary Table S1. Primary antibodies used for immunohistochemistry**

| **Marker** | **Clone / product** | **Manufacturer** | **Catalogue no.** | **Working dilution** |
| --- | --- | --- | --- | --- |
| CD4 | SP35 | Ventana/Roche | 790-4423 | Ready-to-use (RTU) |
| CD8 | SP57 | Ventana/Roche | 790-4460 | Ready-to-use (RTU) |
| CD20 | L26 | Ventana/Roche | 760-2531 | Ready-to-use (RTU) |
| CD155 (PVR) | EPR23074-270 | Abcam, Cambridge, UK | ab267389 | 1:500 |
| Ki-67 | 30-9 | Ventana/Roche | 790-4286 | Ready-to-use (RTU) |
| ER | SP1 | Ventana/Roche | 790-4324 | Ready-to-use (RTU) |
| PR | 1E2 | Ventana/Roche | 790-2223 | Ready-to-use (RTU) |
| p53 | DO-7 | ZSGB-BIO, Beijing, China | ZM-0408 | Ready-to-use (RTU) |
| HER2 | HercepTest | Dako/Agilent Technologies | SK001 | Ready-to-use kit |
| EGFR | 3C6 | Ventana/Roche | 790-2988* | Ready-to-use (RTU) |
